# Supplementary material for: Diagnosis and Treatment Using Autologous Stem-Cell Transplantation in Primary Central Nervous System Lymphoma: A Systematic Review
Source: Cancers (Basel). 2023 Jan 15;15(2):526. doi: 10.3390/cancers15020526 (PMC9856418; doi:10.3390/cancers15020526)
Supplement: Supplementary file 1 [file cancers-15-00526-s001.zip › cancers-2149117-supplementary.pdf]

Table S1. High dose chemotherapy regimens and autologous stem cell transplantation applied in relapsed/refractory PCNSL patients.

Phase of study [ref]	n. pts (median age)	Salvage therapy	ORR (CRR) before ASCT in PCNSL	n. transplanted pts (%)	Conditioning regimen	follow up (mo)	EFS or PFS (entire population versus transplanted)	OS	TRM in all population	Late neurotoxicity in all population
Retrospective (Soussain C, et al. J Clin Oncol. 2001) [6]	22 (53 yrs)	14 with cytarabine + etoposide; 1 with HD-MTX, 7 none*	59% (36%)	20 (91%)	Bu/TT/Cy (Busulfan/Thiotepa /Cyclophosphamide)	41	PFS 53% at 3 yr	64% vs 60% at 3 yr for entire population vs transplant pts	3%	32%
Prospective (Soussain C, et al. J Clin Oncol. 2008) [7]	43 (52 yrs)	cytarabine + etoposide	47% (35%)	27 (63%)	Bu/TT/Cy	36	EFS 43% vs 58% at 2 yr	45% vs 69% at 2 yr for entire population vs transplant pts	7%	11%$
Retrospective (Soussain C, et al.Haematologica. 2012) [65]	79 (52 yrs)	7 none. 55 with Cytarabine+etoposide, 12 cytarabine plus cisplatinum, 4 with HD-MTX, 1 with ifosfamide	76% (40%)	79 (100%)	Bu/TT/Cy	56	EFS 38% vs 44% at 5 yrs for entire population vs chemosensible pts	51% vs 62% at 5 yrs for entire population vs chemosensible pts	7%	9%£
Retrospective (Welch MR, et al.Leuk Lymphoma. 2015) [8]	8 r/r PCNSL (47%) (53yrs) or 9 SCNSL (47 yrs)(53%)	All 8 PCNSL pts received MPV±R ± HD-cytarabine	100% (100%)	15 (88%)	Bu/TT/cy	34	3 yrs PFS 93% in all population 100% in PCNSL subgroup	3 yrs OS 93% in all population 100% in PCNSL subgroup	0%	No data of neurotoxicity was reported
Retrospective (Cote GM, et al, Biol Blood Marrow Transplant. 2012) [50]	14 r/rPCNSL, 2 newly diagnosed PCNSL (49yrs); 16 r/r SCNSL (45 yrs)	For PCNSL: 8 with HD-MTX based chemotherapy, 10 temozolomide-based Cchemotherapy, 2 WBRT , 11 received Rituxumab	93% (44%)	16 (100%)	Bu/TT/cy	12	1 yr PFS 90% for all population	1 yr OS 93% for all population	3%	3% (1 case previously treated with WBRT)
Retrospective (Choi MK, et al. In J Hematol. 2013) [9]	45 (57 yrs)	ICE/D or HD-MTX	84% (51%)	18 (40%)	Bu/TT	53	40% at 5 yrs in ASCT pts vs 20% in non-ASCT pts	40% at 5 yrs in ASCT pts vs 30% in non-ASCT pts	0%	Data not available
Retrospective (Bonm AV, et al. J Neurooncol. 2021) [66]	66 (62yrs)	HD-MTX based CT	87% (60%)	11 (17%)	Data not available	40.5	mPFS 25 mo vs 16mo who are not consolidate with ASCT	mOS of all population was 14 mo, that was longer in ASCT pts	Data not available	Data not available

ORR, overall response rate; CRR, complete response rate; ASCT, autologous stem cell transplantation; PCNSL, primary central nervous system lymphoma; EFS, event free survival; PFS, progression free survival; TRM, treatment relate mortality; r/r, relapsed/refractory; SCNSL, secondary central nervous system lymphoma;; WBRT, whole brain radiotherapy; OS, overall survival; HD, high dose; MTX: methotreaxate; ICE/D: ifosfamide, carboplatin, etoposide, and dexamethasone; MPV+R: methotrexate, procarbazine, vincristine, rituximab

*One patient (patient 4) who failed to achieve her third CR with high-dose MTX was given three cycles of R, carmustine and etoposide combined with intraocular R. One patient (patient 13) received R-CHOP, high-dose cytarabine and IT cytarabine

$2 out 5 had received first-line cranial radiotherapy before HDC/ASCT

£57% (4/7) had previously received cranial irradiation

Table S2. Studies assessing the outcome of high dose chemotherapy/autologous stem cell transplantation in newly diagnosed PCNSL patients.

Type of study, n. of patients (median age) [ref]	Induction chemo therapy	Conditioning regimen	n. transplanted pts (%)	ORR (CRR) before HDchemotherapy/ASCT → CRR after ASCT	follow up (mo)	EFS or PFS transplanted pts)	OS transplanted pts	TRM
Retrospective, n=28 (53 yrs) (Abrey LE, et al. J Clin Oncol. 2003) [37]	HD-MTX x 4 cycles-> HD cytarabine x2 cycles	Carmustine/etoposide/Arac/melphalan (BEAM)	14/28 (50%)	14/28 50%, (29%)->8/14, 57%	28mo	2yrs EFS 43%	2 yrs OS 60%	none
Phase II n=25 (51 yrs) (Colombat P, et al Bone Marrow Transplant. 2006) [38]	HD-MTX/ etoposide/ carmustine/methilprednisolone x2 cycles->ifosfamide/HD cytarabine x1 cycle	BEAM (WBRT 30 Gy after ASCT)	17/25 (68%)	21/25 84% (44%)-> 16/17, 94%	34 mo	4yrs EFS=46%	4yrs OS 64%	1/17 (6%)
Retrospective n=6 (53 yrs) (Brevet M, et al. Eur J Haematol. 2005) [39]	HD-MTX/carmustine/etoposide/ methilprednisolone(MBVP)+it Arac/MTX/MPN	BEAM (WBRT 30 Gy after ASCT)	6/6	6/6 100% (2/6, 33%)-> 6/6, 100%	42 mo	NR	mOS 35 mo	none
Phase II n=13 (54 yrs) (Miyao K et al, Int J Hematol. 2014) [42]	HD-MTX-based chemotherapyx3 cycles->HD-cytarabine x2 cycles	Melphalan/ cy/etoposide/dexamethasone (LEED)	6/13 (46%)	13/13 100% (0/13, 0%) > 5/6 83%	44 mo	3-year PFS 83%	3-year OS 80%	none
Phase II n=11 (52 yrs) (Yoon DH, et al Bone Marrow Transplant. 2011) [46]	HD-MTX x5 cycles-> HD- ctarabine x2 cycles	Busulfan/cy/etoposide (BUCYE)	11/11 (100%)	11/11, 100% (8/11, 73%)-> 10/11, 91%	25 mo	mEFS 15 mo	2yrs OS 89%.	none
Phase II n = 32 (57 yrs) (Omuro A, et al Blood 2015) [47]	Rituximab/HD-MTX/ vincristine/ procarbazine (RMVP)	Bu/TT/Cy (Busulfan/Thiotepa/Cyclophosphamide) (WBRT after ASCT in 2 pts)	26/32 (81%)	30/32, 94% (21/32, 66%) → 21/26, 81%	45 mo	2-year PFS 81%	2-year – 81%	3/26 (12%)
Phase II n=21 (56 yrs) (Alimohamed N,et al. Leuk Lymph. 2012)[48]	HDMTX/cytarabinebased chemotherapy	Bu/TT /Cy	21/21 (100%)	18/21 ,86% (5/21, 24%)->11/21,52%	60 mo	5-yrs PFS 44%; 5-yrs TTP 65%	5-yrs OS 44%	3/21 (14%)
Retrospective n=16 (49 yrs) (Cote GM, et al. Biol Blood Marrow Transplant. 2012) [50]	HD MTX-temozolomide based chemotherapy	Bu/TT /Cy	16/16 (100%)	15/16, 94% (7/16, 44%)->NR	379 days	1-yrs PFS 90%	1 yrs OS 93%	0/16
Phase II n=18 (54 yrs) (Chen YB, et al. Cancer. 2015) [51]	HD MTX+Rituximab based-chemotherapy	HD Rituximab/ Bu/TT /Cy	18/18 (100%)	18/18, 100% (16/18, 89%)->NR	24 mo	2yrs PFS 100%	2yrs OS 100%	0/18
Phase II n=23 (55 yrs) (Montemurro M, et al. Ann Oncol. 2007) [52]	HD MTX	busulfan/thiotepa (WBRT after ASCT in 9 pts)	16/23 (70%)	23/23, 100% (70%)->11/16,69%	15 mo	2 yrs EFS 56%	2 yrs OS 61%	2/16 (13%)
Phase II n=30 (54 yrs) (Illerhaus G, et al. JCO 2006) [53]	3x HD MTX->2x cytarabine/thiotepa	Carmustine/ thiotepa (5 mg/kg BID)-> hyper fractionated WBRT	23/30 (77%)	24/30, 80% (10/30, 33%)->15/23, 65%	63 mo	5-year relapse-related death 8.7%	5yrs OS 87%	0/23 (0%)
Phase II n=43 (54 yrs)	HD MTX based chemotherapy	BCNU/Thiotepa (2 × 5 mg/kg in 23 pts; 4 × 5 mg/kg in 11 pts)	34/43 (79%)	NR->27/34,79%	120 mo	5-year EFS 79%	5-year OS 82%	0/34

(Kasenda B, et al. Ann Oncol 2015) [54]								
Retrospective n=45 (60 yrs) (Wullenkord R, et al. Ann Hematol 2021) [29]	NR	Thiotepa-based chemotherapy	45/45 (100%)	43/45, 96% (15/45, 33%)-> 35/45,78%	25 mo	mPFS 63 mo	mOS 65 mo	0/45 (0%)
Phase II randomized (consolidation with WBRT n=70 (54yrs) vs HDC/ASCT n=70 (55yrs) (Houillier C, et al. JCO 2019) [16]	R-MBVP (Rituximab, Methotrexate, Carmustine, Etoposide, and Prednisone) → Rituximab/ HD-cytarabine	Bu/TT/Cy	44/70 (63%)	ORR 70% (CR 43%) in both arm; ORR 64% (CR 38%) in arm B → NR	33 mo	2-year – 87%	2-year – 66%	5/44 (11.4%)
Phase II randomized: consolidation with WBRT n=59 (58 yrs) vs HDC/ASCT n=59 (58 yrs) (Ferreri AMJ, et al Lancet Haematol 2017) [17]	HD-MTX/HD-cytarabine/±Rituximab±Thiotepa	Carmustine/thiotepa (5mg/kg BID)	54/59 (92%)	ORR 118/227:52% (63/227,28%) before randomization → 55/59: 93% of arm HD chemotherapy/ASCT	40 mo	2-year PFS–69%	2-year – 77%	2/54 (4%)
Phase II randomized (consolidation with HDC/ASCT (n=54) vs non-myeloablative chemotherapy (n=54) n=113 (61 yrs) (Batchelor T, et al. JCO 2020) [60]	HD MTX/temozolomide/cytarabine/rituximab (MTRA)	Carmustine/ Thiotepa (5 mg/kg BID, D -5, -4)	36/54 (67%)	NR	45.6 mo	mPFS 6 yrs	3 yrs OS 83%	0/36 (0%)
Phase II randomized (consolidation with HDC/ASCT (n=114) vs non-myeloablative chemotherapy (n=115) (59 yrs) (Illerhaus G et al. Blood 2022)	HD-MTX/HD-cytarabine/Thiotepa/Rituximab	Carmustine/ Thiotepa (5 mg/kg BID)	114/260 (44%)	69%	44 mo	3-years PFS 79%	3 years OS 86%	4/114

ORR, overall response rate; CRR, complete response rate; HDC/ASCT, high dose chemotherapy/autologous stem cell transplantation; PCNSL, primary central nervous system lymphoma; EFS, event free survival; PFS, progression free survival; TRM, treatment relate mortality; WBRT, whole brain radiotherapy; OS, overall survival; HD, high dose; MTX, methotrexate; ; NR, not reported
